# The Role of Transport Mechanisms in *Mycobacterium Tuberculosis* Drug Resistance and Tolerance

**DOI:** 10.3390/ph5111210

**Published:** 2012-11-09

**Authors:** Jansy Passiflora Sarathy, Véronique Dartois, Edmund Jon Deoon Lee

**Affiliations:** 1 Novartis Institute for Tropical Diseases Pte Ltd, 10 Biopolis Road #05-01, Chromos, 138670, Singapore; 2 Department of Biological Sciences, National University of Singapore, 14 Science Drive 4, 117543, Singapore; 3 Department of Pharmacology, Yong Loo Lin School of Medicine, National University of Singapore, Clinical Research Centre, 10 Medical Drive, 117597, Singapore

**Keywords:** *Mycobacterium tuberculosis*, drug transport, efflux, porins, resistance, persistence

## Abstract

In the fight against tuberculosis, cell wall permeation of chemotherapeutic agents remains a critical but largely unsolved question. Here we review the major mechanisms of small molecule penetration into and efflux from *Mycobacterium tuberculosis* and other mycobacteria, and outline how these mechanisms may contribute to the development of phenotypic drug tolerance and induction of drug resistance. *M. tuberculosis* is intrinsically recalcitrant to small molecule permeation thanks to its thick lipid-rich cell wall. Passive diffusion appears to account for only a fraction of total drug permeation. As in other bacterial species, influx of hydrophilic compounds is facilitated by water-filled open channels, or porins, spanning the cell wall. However, the diversity and density of *M. tuberculosis* porins appears lower than in enterobacteria. Besides, physiological adaptations brought about by unfavorable conditions are thought to reduce the efficacy of porins. While intracellular accumulation of selected drug classes supports the existence of hypothesized active drug influx transporters, efflux pumps contribute to the drug resistant phenotype through their natural abundance and diversity, as well as their highly inducible expression. Modulation of efflux transporter expression has been observed in phagocytosed, non-replicating persistent and multi-drug resistant bacilli. Altogether, *M. tuberculosis* has evolved both intrinsic properties and acquired mechanisms to increase its level of tolerance towards xenobiotic substances, by preventing or minimizing their entry. Understanding these adaptation mechanisms is critical to counteract the natural mechanisms of defense against toxic compounds and develop new classes of chemotherapeutic agents that positively exploit the influx and efflux pathways of mycobacteria.

## Abbreviations

ABC: ATP-Binding Cassette transporters
MFS: Major Facilitator Superfamily transporters
RND: Resistance-Nodulation-Cell Division transporters
ATP: Adenosine triphosphate
CCCP: Carbonyl cyanide m-chlorophenyl hydrazone
PMF: Proton Motive Force

## 1. Introduction

In 2010, it was estimated that there were 8.8 million incident cases of tuberculosis (TB), 1.1 million deaths from TB among HIV-negative people and an additional 0.35 million deaths from HIV-associated TB [1]. Despite the availability of effective treatment options since the 1950s, and the implementation of well-structured treatment programs, the current TB epidemic is not being controlled. Frontline anti-tuberculous drugs have gradually become ineffective because of the increasing incidence of resistance. Multidrug-resistant TB (MDR-TB) is a difficult-to-treat form of *M. tuberculosis* that fails to respond to the two most effective first-line anti-tuberculous drugs, rifampicin and isoniazid. The World Health Organization (WHO) estimated that in 2009, around 5% of all new tuberculosis cases involved MDR-TB [2]. Strains that combine MDR with additional resistance to fluoroquinolones and at least one injectable drug have been appropriately named extensively drug-resistant tuberculosis (XDR-TB). The burden of tuberculosis on global health has pushed the research community into focusing efforts on the development of new vaccines, diagnostics and chemotherapy against *Mycobacterium tuberculosis*, the causative agent.

**Figure 1:**
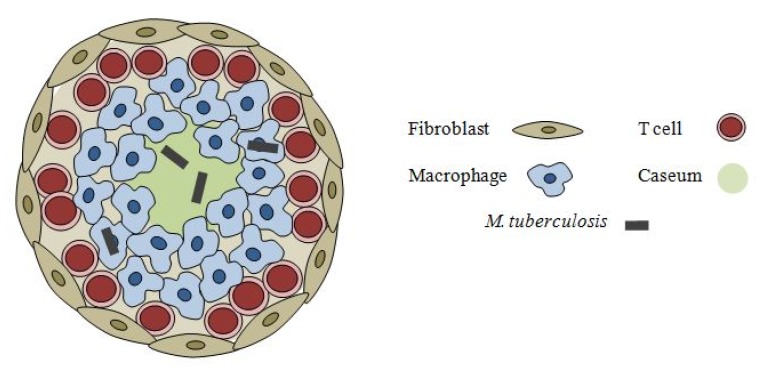
illustrates a classic tuberculous granuloma with a caseous centre that can be found in both actively- and latently-infected patients. *M. tuberculosis* in such granuloma can be found intracellularly within macrophages or extracellularly.

The TB pathology is diverse, generating different types of lesions, containing several micro-environments each harboring metabolically distinct bacterial sub-populations, some of which are not effectively killed by most existing drugs [3]. This drug tolerance phenomenon typical of tuberculosis has been coined “phenotypic drug resistance” [4], and is partly attributed to the pathogen’s ability to remain sequestered in macrophages and other stress-inducing micro-environments in a non-replicating state of persistence (see Figure 1) [5]. These dormant bacilli are primarily responsible for the persistent and latent forms of the disease, but retain the potential to resume growth and produce an active infection, making them a critical target population of antimycobacterial agents [5,6,7].

The development of new antimycobacterials active against dormant cells and resistant strains is in need of novel drug targets. The failure of existing chemotherapeutic options to control the TB epidemic can be attributed in part to sub-therapeutic concentrations at the site of action [8]. The longer a pool of bacteria is exposed to sub-inhibitory levels of an antimicrobial agent, the more likely the emergence and selection of resistant clones becomes [9]. This has prompted researchers and drug discovery experts to turn to strategies which would potentiate existing therapeutics by increasing their intracellular levels through the use of small molecule inhibitors against efflux pumps [10].

The cell envelope of mycobacteria is notorious for being several-fold less permeable to chemotherapeutic agents when compared to functionally similar cell walls of other bacteria [11]. The knowledge of drug transport pathways could assist in the successful design of novel chemotherapeutic combinations against *M. tuberculosis*. Here, we review the current understanding of the various influx and efflux pathways in mycobacteria while focusing our attention on details specific to *M. tuberculosis*. The function and expression of transport proteins such as porins, drug importers and efflux pumps are summarized and their respective influence on the drug-resistant and non-replicating persistent states is highlighted. Collectively, the literature data compiled here show that *M. tuberculosis* and other mycobacteria have evolved several intrinsic and adaptive mechanisms to increase their level of tolerance towards xenobiotic substances, by preventing or minimizing their entry: (i) natural or intrinsic resistance mediated by the thickened highly hydrophobic and waxy envelope; (ii) reduced permeability resulting from physiological adaptations under unfavorable environmental conditions; (iii) drug-induced resistance acquired via increased expression of various classes of efflux pumps; and (iv) genetically encoded resistance conferred by mutations in efflux complexes. 

## 2. Mycobacterial Cell Wall: The Permeability Barrier

The cell envelope of mycobacteria is structurally distinct from that of both Gram-positive and Gram-negative bacteria. The entire mycobacterial cell envelope can be broken down into two main structural components: cell membrane and cell wall. The outer leaflet of the cell wall is composed of mycolic acids which are covalently linked to the arabinogalactan-peptidoglycan complex of the inner leaflet. Mycobacteria are capable of producing a multitude of mycolic acids with varying lengths and modifications depending on species, strain and growth conditions [12,13,14]. It is widely believed that the unusually high mycolic acid content, combined with a variety of other intercalated lipids, contributes to the wall’s limited permeability [15]. The mycobacterial cell wall is also composed of phosphotidyl-*myo*-inositol derived glycolipids such as lipomannan and lipoarabinomannan which have potent immunomodulatory activities [16].

The mycolyl-arabinogalactan-peptidogalactan complex is acknowledged as being a more efficient permeability barrier than cell walls of any other class of bacteria [11]. Jarlier and Nikaido attempted to clearly define the mycobacterial permeability barrier to hydrophilic molecules by studying the uptake kinetics of small nutrient molecules (glucose, glycine, leucine and glycerol) in *M. chelonae* [17]. The permeability coefficients (P) for these nutrients were found ranging from 1.4 to 62 nm/s; specifically 2.8 nm/s for glucose. K_m_ values of the overall transport of glucose and glycerol were 1,000 µM and 200 µM, respectively, as measured in the same study. In comparison, a different study had measured a permeability coefficient of glucose for *E. coli* (1.4 × 10^5 ^nm/s) that was about five orders of magnitude higher [18]. It should be noted that the precise values of permeability differ among different species of mycobacteria. *M. chelonae*, being one of the most drug-resistant species, has a cell wall that is about one to two orders of magnitude less permeable than *M. tuberculosis*, *M. smegmatis* and *M. phlei* [11,18]. This intra-species difference in cell wall permeability may be attributed to variability in its content and organization. Detailed structural and quantitative analysis has revealed a higher mycolate-to-peptidoglycan ratio in *M. leprae* than *M. tuberculosis*; peptidoglycan coverage by mycolate was estimated at 80% and 63% for *M. leprae* and *M. tuberculosis*, respectively [19].

This unique cell wall composition and organization is believed to render mycobacteria less susceptible than other bacterial pathogens to various antibiotic classes [11,20]. Several pathways exist for compounds to cross this permeability barrier. It is assumed that hydrophobic compounds should be able to penetrate cell walls by simply dissolving into and through the lipophilic cell wall unassisted, whereas the influx of hydrophilic compounds is largely facilitated by porins, which are water-filled open channels that span the cell wall [21]. It appears that the mycobacterial plasma membrane plays a limited role in pathogenicity and maintenance of the influx-efflux equilibrium [13,22].

## 3. Passive Diffusion: The Hydrophobic Pathway

In principle, antimicrobial agents of the more lipophilic classes such as the rifamycins, macrolides and fluoroquinolones are more likely to diffuse into and through the lipid-rich environment of the mycobacterial cell wall in order to transverse its depth [20]. This passive transport has been coined “hydrophobic (or lipid) pathway”, characterized by the nature of the interactions between structural lipids and small molecules [23]. However, lipophilic agents are presumably slowed down by the low fluidity and unusual thickness of the cell wall [24]. It has been demonstrated that lipophilic derivatives within single drug classes are more active against mycobacteria when compared to their hydrophilic counterparts [20]. This was more recently supported by evidence from a comparison of Minimum Inhibitory Concentrations (MIC) between hydrophilic and hydrophobic fluoroquinolone analogs. Moxifloxacin (cLogP 0.6) was 32-fold more effective than norfloxacin (cLogP −0.1) at inhibiting the growth of *M. smegmatis* [25], though the relative affinity of fluoroquinolones for the gyrase and differential susceptibility to efflux pumps also contribute to the net difference in MIC. Brennan *et al.* postulated that an increase in the rate of drug penetration resulting from an increase in incubation temperature is also evidence of the predominant role of the hydrophobic pathway or passive diffusion in drug penetration [20].

## 4. Facilitated Diffusion: The Porins

Porins are large water-filled channels allowing the penetration of small hydrophilic molecules without requiring energy consumption. This uptake pathway caters to a limited range of compounds since channel diameters at the narrowest point define the exclusion limit, and parameters such as channel length and the number of open pores determine the velocity of transport [26]. As demonstrated by studies in *E. coli*, diffusion rates through porins are further affected by the charge, hydrophobicity, and size of the solute [21,27,28]. Several types of porins have been identified and studied in Gram-negative and some Gram-positive bacteria. To date, two putative classes of porins have been identified and characterized in mycobacteria; they are MspA-like and OmpA-like porins in *M. smegmatis* and *M. tuberculosis* respectively [25].

MspA was the first porin of its class identified in a mycobacterial species, with proven oligomerization and channel-forming activity *in vitro* and when cloned in *E. coli* [29]. Subsequent sequencing of the *M. smegmatis* genome revealed three more porin genes with homology to *mspA*, namely *mspB*, *mspC* and *mspD* [30]. Numerous studies have documented MspA-enabled transport of hydrophilic solutes and drug molecules across the cell wall of *M. smegmatis*. Table 1 summarizes drug transport specificities of various *M. smegmatis* porins and their impact on drug uptake and MIC. The studies show that porin deletion is clearly linked to increases in MICs of various antibiotics. In several instances, this increase in MIC has been associated with reduction in drug uptake. Furthermore, heterologous expression of *M smegmatis mspA* accelerated the growth and increased the susceptibility of *M. tuberculosis* and *M. bovis* BCG to various classes of antibiotics [23]. This establishes the relationship between porin function, small molecule and nutrient uptake, and drug susceptibility in *M. smegmatis.*

**Table 1:** Specific drug transport activities of mycobacterial porins of the Msp class in *M. smegmatis*. In all instances, porin-deletion mutants were used to determine drug transport specificity; dependence of individual drugs on porin transport is exemplified by the extent of reduction in drug uptake and increase in MIC.

Species	Deleted Porin	Drug	Fold-reduction in Drug Uptake	Fold-increase in MIC	Reference
***M. smegmatis***	MspA & C double deletion	Ampicillin	-	16	[25]
		Cephaloridine	-	8	
		Chloramphenicol	1–2	4	
		Norfloxacin	4	2	
	MspA	Ampicillin	-	16	[31]
		Cephaloridine	9	8	
		Vancomycin	-	10	
	MspA	Cephaloridine	9	-	[30]

Porins are only minor proteins in the mycobacterial cell wall unlike enterobacterial porins which are the most abundant proteins in the cell [23,26]. Direct counting of stained pores by electron microscopic analysis revealed a 45-fold lower number of MspA pores on the outer membranes of *M. smegmatis* when compared to pore counts of the outer membranes of Gram-negative bacteria [32]. The octameric MspA porin consists of two consecutive hydrophobic β-barrels, a more hydrophilic globular rim domain, and a single central channel of 9.6nm in length. This porin is largely embedded into the cell membrane of *M. smegmatis* with the embedded region including a portion of the hydrophilic rim domain [33]. Crystal structures revealed that the constriction zone of MspA is rich in aspartate residues. Together with the high number of negative charges in the vestibule and channel interior, this could explain the cation preference of MspA [34].

OmpATb was the first suggested porin-like molecule identified in *M. tuberculosis.* Encoded by the *rv0899* gene, the name OmpATb was coined because of its homology to the *E.coli* porin OmpA [35].A study by Teriete *et al.* showed that Rv0899 does not form a transmembrane β-barrel, but a mixed α/β globular structure encompassing two independently folded modules which correspond to the B and C domains of the protein. The core of this B domain appears hydrophobic while its exterior is both polar and acidic [36]. Altogether, this proposed structure for OmpATb makes it unlikely for it to function as a porin. More recent structural elucidation by Yang *et al.* suggests that OmpATb forms a heptameric ring complex, driven by interactions between the α/β structured monomers, and is hypothetically capable of inserting itself into a biological membrane and form channels [37], allowing ion diffusion as observed *in vitro*. This model is based on NMR data of minor oligomeric populations of OmpATb in solution, and lies in contrast with available data suggesting the lack of functional porin assembly *in vitro* [38].

OmpATb plays a key role in conferring *M. tuberculosis* the ability to survive under acidic conditions. Deletion mutants in *ompATb* exhibit a significant reduction in permeability to several hydrophilic molecules and impaired ability to grow at reduced pH. The role of OmpA in acid resistance was reinforced by the observation of increased *ompATb* transcription levels in *M. tuberculosis* growing within macrophages, given that vacuole acidification is known to occur in infected phagocytes [39]. More recent functional studies revealed no obvious porin activity of OmpATb. Rather, chemical analysis of low-pH *M. tuberculosis* culture filtrates showed that OmpATb is involved in rapid ammonia secretion capable of neutralizing medium pH and restoring exponential bacterial growth. This is further substantiated by the discovery that Rv0899-like proteins are present predominantly in bacteria with functions in nitrogen fixation and metabolism [40]. OmpATb-mediated ammonia extrusion may be one of the multiple adaptations of *M. tuberculosis* to acidic environments and, on its own, is not critical for virulence in mice [38]. The porin function of OmpATb in *M. tuberculosis* clearly remains a controversial issue.

A recent attempt to predict outer membrane proteins in *M. tuberculosis* via a bioinformatics approach has led to the identification of two novel Omp-like proteins (Rv1698 and Rv1973) in *M. tuberculosis.* Both proteins have been proven to localize to the outer membrane [41]. Since then, the channel-forming activity of Rv1698 has been successfully characterized; *Rv1698* expression has proven to restore sensitivity of MspA-deletion mutants of *M. smegmatis* to ampicillin and chloramphenicol, and complement the permeability defect of the mutant for glucose. Single homologues of Rv1698 are found only in mycolic-acid containing bacteria belonging to the suborder Corynebacterineae of the Actinomycetales, which includes mycobacteria. It therefore represents the first protein identified as specific for this suborder [42]. Orthologous porins PorM1 and PorM2 have since been characterized in *M. fortuitum* [43].

Interestingly, *M. tuberculosis* does not express the Msp-like porins that are found in faster-growing *M. smegmatis* [32]. This and other significant differences between the pathogenic and saprophytic mycobacterial species call into question the appropriateness of *M. smegmatis* as a model organism for anti-tuberculosis drug discovery and virulence studies of *M. tuberculosis* [15]. 

Table 2 summarizes several biophysical characteristics of mycobacterial and other bacterial porins. Single-channel conductance often provides an estimation of channel diameters of porins, and gives an indication of the relative mobilities of solutes through them. It can be observed from Table 2 that some proportionality exists between channel width, channel conductance and size exclusion limits. If OmpATb of *M. tuberculosis* does indeed form functional porin units, this trend would place its limit in the approximate range of 600–800 Da. This implies that the rifamycin and macrolide classes are too large to utilize OmpATb to transverse the cell wall. Danilchanka *et al* attempted to illustrate the fit of a drug when oriented along their longest axes within the MspA porin constriction zone by using 3D structure visualization and surface representations of structural models of antibiotics. They predicted that ampicillin, chloramphenicol and norfloxacin are able to utilize this porin molecule, as opposed to antibiotics such as erythromycin, kanamycin and vancomycin [25].

**Table 2:** Biophysical characteristics of OmpATb from *M. tuberculosis* and porins from other selected bacterial species. Exclusion limits were determined based on the uptake of saccharides of varied weight.

Species	Porin	Channel Width (nm)	Single-Channel Conductance (nS)	Exclusion Limit (Da)	Reference
***M. tuberculosis***	OmpATb	1.4–1.8	0.7	Undetermined	[35]
***M. smegmatis***	MspA	2.2–2.4	4.6	Undetermined	[44,45]
***E. coli***	OmpA	0.6–0.7	0.14 (at 37 °C)	550*	[46,47,48]
	OmpF	1.2	0.82		
***P. aeruginosa***	OprF	2.2	5	6000	[49,50]
***S. typhimurium***	Not specified	1.4	2.3	700	[46,51]

* This study on size exclusion limit for *E.coli* porins did not distinguish between specific Omp types.

The specific role played by porins in intracellular drug accumulation within other bacterial species has been well studied. The outer membrane porin protein OprD of *Pseudomonas aeruginosa* has been directly implicated in the influx of imipenem; a staggering 98% of imipenem- and meropenem-resistant *P. aeruginosa* clinical isolates have been identified as being negative for OprD porin production [52]. Similarly, studies on expression levels of the porin protein OmpF in clinical isolates of *E. coli* have linked the decreased expression levels of this porin with resistance to quinolones [53,54]. In these enterobacterial pathogens, the reduction in the number of functional porins per cell is mostly due to a decrease or complete shutdown of synthesis, or the expression of an altered porin. These changes bring about decreased susceptibility to antimicrobials and favor the acquisition of additional mechanisms of bacterial resistance [55]. Changes in the expression levels of functional porins should therefore be viewed as potential contributing factors in the development of resistance in mycobacteria. 

In conclusion, porins appear to be less varied and less abundant in mycobacteria than in other bacterial families such as the enterobacteriaceae, though the possibility remains that we have only detected a small fraction of the total mycobacterial porin panel. The wide range of metabolic and physiologic adaptations seen in *M. tuberculosis*, combined with the generally complex regulation of porin expression in other species, suggest that *M. tuberculosis* has likewise exploited porin modulation as a strategy to fence itself off from harmful small molecules. Some of these putative schemes are discussed in a later section.

## 5. Active Transport Processes: Influx and Efflux

### 5.1. Influx Transporters

Based on *M tuberculosis* genome sequence analysis, Braibant *et al.* have concluded that there is an under-representation of importers in *M. tuberculosis*, with the exception of phosphate importers, when compared to other bacterial species such as *E. coli* and *B. subtilis* [56]. In addition, the ratio of exporter-to-importer proteins, based on sequence homology, is markedly higher in *M. tuberculosis* than in *E. coli.* This observation may again contribute to the reduced uptake of small molecules by *M. tuberculosis* bacilli. Though bacterial ABC transporters can mediate both influx and efflux activity, only their efflux activity has been observed and characterized in mycobacterial species [57]. Identified substrates for ABC influx activity thus far include sugars, amino acids, metals and anions [58].

### 5.2. Efflux Pumps

#### 5.2.1. Resistance Phenotype I—Natural Abundance

The presence of active multi-drug efflux pumps is also thought to play a significant role in the development of natural and induced drug resistance in mycobacteria. In 1998, the complete genome sequencing of *M. tuberculosis* revealed at least 14 members of the Major Facilitator Family (MFS) and the ATP-binding Cassette (ABC) transporter family [59]. In 2000, analysis of transcriptional clusters and homology searches of transporters from other organisms allowed for the reconstitution of 26 complete and 11 incomplete ABC transporters from the various subunits encoded for by the complete *M. tuberculosis* genome [56]. In the same study, it was concluded that ABC transporters account for 2.5% of the genome of *M. tuberculosis*. This compares with 5% of the entire *E. coli* genome that encodes for 69 ABC transporters [60]. ATP-binding cassettes (ABC), the major facilitator superfamily (MFS), the multidrug and toxic compound extrusion (MATE) family, the small multidrug resistance (SMR) family and the resistance-nodulation-division (RND) superfamily are the five families of bacterial drug efflux pumps that have been categorized thus far [61,62,63]. The mechanisms of efflux-mediated drug resistance in bacteria have been well-studied and reviewed over the past decade, and are therefore only briefly summarized here.

ABC transporter proteins are known for coupling ATP-hydrolysis with the alternation between outward- and inward-facing conformations to bring about substrate transport [64]. MFS and RND transporters, on the other hand, are classified as secondary active transporters because they are driven by the proton-motive force (PMF) [65]. SMR transporters are the smallest multidrug resistant proteins, with lengths of about a 110 amino acids only. Despite the general correlation between genome size and the number of ABC transport systems, the *M. tuberculosis* genome encodes fewer ABC systems per megabase than any other organism surveyed in a comprehensive analysis of the solute transport systems within the genomes of 18 prokaryotes. It was suggested that the relative abundances of ABC and MFS transporters reflects the overall use of energy coupling mechanism in each organism. *M. tuberculosis*, being a strict aerobe, is more dependent on PMF-type secondary transporters as compared to fermentative organisms that depend on substrate level phosphorylation to generate ATP. Also worth noting is the largest RND-family representation in *M. tuberculosis* compared to the other prokaryotes surveyed. These are believed to play a significant role in the extrusion of lipids and other cell envelope components [66].

Several ABC, MFS, RND and SMR efflux pumps of *M. tuberculosis* and other mycobacteria have been characterized as antibiotic transporters (See Table 3). TetV and LfrA, which have been identified in *M. smegmatis* as drug transporters but not in *M. tuberculosis* have also been included in the table. Some of these putative pumps have been associated with reduced mycobacterial susceptibility to agents such as isoniazid, tetracycline, fluoroquinolones and aminoglycosides [67]. Differences in efflux pump expression between mycobacterial species are important because they offer insights into the acquisition of drug resistance. One study which investigated influx and efflux rates of pyrazinamide and pyrazinoic acid, respectively, revealed that the efflux rate in *M. smegmatis* is 900 fold higher than in *M. tuberculosis* when no significant variability was noticed in uptake rates. It is not known whether this difference is due to variability in the type or expression level of pumps present in both species but it potentially explains the innate resistance of *M. smegmatis* to pyrazinamide as compared to the relative susceptibility of *M. tuberculosis* [68].

**Table 3:** Summary of known antibiotic substrates of several mycobacterial efflux pumps of *M. tuberculosis.*

Pump	Gene	Transporter Family	Known Substrates	Known Inhibitors	Energy Source	Mycobacteria	Reference
-	*rv2686c-*	ABC	Fluoroquinolones	Verapamil	ATP	*M. tuberculosis*	[69]
	*rv2687c-*			Reserpine			
	*rv2688c*			CCCP			
-	*rv1218c*	ABC	Novobiocins	Verapamil	ATP	*M. tuberculosis*	[70]
			Pyrazolones	Reserpine			
			Pyrroles	CCCP			
-	*rv0194*	ABC	Ampicillin	Reserpine	ATP	*M. tuberculosis*	[25]
			Chloramphenicol				
			Streptomycin				
			Novobiocin				
**DrrAB**	*drrA-drrB*	ABC	Doxorubicin	Verapamil	ATP	*M. tuberculosis*	[71]
				Reserpine			
**MmpL7**	*mmpL7*	RND	Isoniazid	ReserpineCCCP	PMF	*M. tuberculosis*	[72]
**Tap**	*rv1258c*	MFS	Tetracycline	Piperine	PMF	*M. tuberculosis*	[73,74,75]	
			Rifampicin			*M. fortuitum*	
**P55^b^**	*rv1410c*	MFS	Rifampicin	CCCP	PMF	*M. tuberculosis*	[76,77]
			Clofazimine				
			Aminoglycosides	Valinomycin		*M. bovis*	
			Tetracycline				
**JefA**	*rv2459*	MFS	Isoniazid	Verapamil	Not speculated	*M. tuberculosis*	[78]
			Ethambutol				
				CCCP			
			Streptomycin				
**EfpA**	*rv2846c*	MFS	Not determined	-	PMF	*M. tuberculosis*	[67,79]
						*M. smegmatis*	
						*M. leprae*	
						*M. avium*	
**IniA^a^**	*iniA*	-	Isoniazid	Reserpine	Not speculated	*M. tuberculosis*	[80]
			Ethambutol				
**Mmr**	*rv3065*	SMR		CCCP	PMF	*M. tuberculosis*	[81,82]
			Erythromycin				
			Thioridazine				
**Tet(V)**	*tet(V)*	MFS	Tetracycline	CCCP	PMF	*M. smegmatis*	[81]
						*M. fortuitum*	
**LfrA**	*lfrA*	MFS	Fluoroquinolones	CCCP	PMF	*M. smegmatis*	[83]
			Doxorubicin				

^a^ IniA is itself a pump component that hypothetically participates in the formation of a multimeric structure with a central pore. ^b^ The function of P55 is connected to P27, a proposed glycolipid transporter [84]. Both proteins are encoded in the IprG-Rv1410c operon of *M. tuberculosis* [85].

#### 5.2.2. Resistance Phenotype II—Induction of Expression

Studies have shown that the exposure to various anti-tuberculous drugs can trigger increased expression of selected efflux pumps leading to drug-mediated phenotypic resistance. Two possible mechanisms are thought to contribute to higher expression of pump-encoding genes: transitory induction by the substrate of these pumps and mutations in the promoter and regulatory region leading to increased or constitutive expression [67,86]. The latter is discussed in the next section. The study of time-kill kinetics of isoniazid against wild-type *M. tuberculosis* revealed that while rapid concentration-dependent killing was seen upon initial drug exposure, subsequent re-growth was observed over a wide range of isoniazid concentrations which was caused by the development of isoniazid-resistant sub-populations. Susceptibility of this subpopulation to isoniazid was restored in the presence of an efflux pump inhibitor for 98% of the resistant clones [87], suggesting that the majority of the isoniazid-resistant population represents efflux pump-mediated phenotypic drug tolerance, though genetic mutations in efflux pump-encoding genes were not formally excluded in this study. More recently, it has been established that susceptible and rifampicin mono-resistant *M. tuberculosis* strains develop resistance to isoniazid within three weeks, and that this is effectively prevented by efflux pump inhibitors [88]. Such induction of resistance to isoniazid has been associated with the overexpression of efflux pump genes such as *mmpl7*, *p55*, *efpA*, *mmr*, *Rv1258* and *Rv2459* [88,89,90]. In the presence of isoniazid, wild-type *M. bovis* BCG and *M. tuberculosis* increased the expression of *iniA* by up to 10-fold [80]. Though it does not appear to directly transport isoniazid out of the cell, this predicted transmembrane protein has been postulated to serve as a pump component that participates in the formation of a multimeric structure containing a central pore.

Gupta *et al.* demonstrated the overexpression of 10 efflux pump genes in MDR strains following exposure to a range of anti-tuberculous drugs. The simultaneous expression of *Rv2459*, *Rv3728* and *Rv3065*, for example, has been associated with resistance to the specific combination of isoniazid and ethambutol, while *Rv2477* and *Rv2209* overexpression has been associated with ofloxacin stress [78]. One MDR clinical isolate bearing defined mutations in *katG* and *rpoB* displayed *rv1258c* and *Rv1410c* overexpression upon rifampicin or isoniazid exposure, and *Rv1819c* overexpression upon isoniazid exposure alone [91,92].

Interestingly, evidence exists for the reduction in susceptibility of *M. tuberculosis* to one drug upon exposure to another. The exposure of rifampicin-resistant strains to rifampicin resulted in a reduction in susceptibility to ofloxacin which could be restored by the introduction of efflux pump inhibitors [93]. One could hypothesize that the up-regulated efflux pumps are promiscuous in their activity and that the cyclic nature of both drugs facilitates recognition by similar pumps.

Finally, induction of selected efflux pumps contributes to drug resistance in biofilms formed by both Gram-positive and Gram-negative bacteria [61], though such phenomenon has not been demonstrated for *M. tuberculosis* to date. Signaling molecules that play a role in cell-to-cell communication are susceptible to efflux pumps, leading to the modulation of inter-species communication in the control of drug resistance and virulence in Salmonella [94]. Development of drug resistance through similar mechanisms remains to be explored in mycobacteria. All these findings indicate that there may be a role for efflux pump inhibitors in the treatment of TB, including latent TB, MDR-TB and XDR-TB.

#### 5.2.3. Resistance Phenotype III—Efflux Pump Mutations

Drug efflux is typically described as an intrinsic or natural resistance mechanism in bacteria. However, mutations in efflux pump genes and their regulator sequences can lead to increased efflux activity and, hence, enhanced drug resistance. Such mutational events either cause an inducible increase in pump expression upon antibiotic exposure, or the constitutive expression of otherwise tightly controlled pump genes above basal levels [95]. Several such mutations have been documented in various bacterial systems, particularly in Gram-negative species [96,97,98]. Often, the mutations are stable point mutations that reduce the DNA binding affinity of particular repressors for their target regulatory region within promoters and lead to constitutive expression of efflux components [95].

In *M. tuberculosis*, mutations in the bioactivating enzymes or in the target of rifampicin, isoniazid, pyrazinamide and the fluoroquinolones cannot explain all clinically observed resistance. For example, approximately 20 to 30% of INH-resistant *M. tuberculosis* isolates do not have mutations in any of the known genes associated with INH resistance [99]. Similarly, approximately 5% of clinical RIF-resistant M. tuberculosis isolates do not harbor mutations in the RIF resistance-determining region of the *rpoB* gene [100]. A number of studies based on gene expression profiling and efflux pump inhibition point towards the role of active extrusion in genotypic drug resistance [57]*.* However, due to incomplete understanding of efflux substrate specificities and regulatory mechanisms, the distinction between expression induction by the substrate resulting in transient tolerance versus DNA mutations leading to inherited up-regulation at the transcriptional level has not been clearly made in most cases. The growing pool of whole genome sequences from clinically resistant isolates provides a unique opportunity to elucidate some of the mechanisms underlying efflux-mediated drug resistance in *M. tuberculosis*.

## 6. Phenotypic Drug Tolerance

### 6.1. The NRP State

Non-replicating persistence (NRP) is defined as the physiological state of bacteriostasis in addition to metabolic, chromosomal and structural changes in the bacilli that enable the conservation of energy [7]. Sufficient evidence has emerged for long-term NRP of *M. tuberculosis* in the human host within tuberculous granulomas and necrotic lesions in pulmonary tissue. Nutrient limitation and hypoxic conditions within granulomas trigger the shutdown in central metabolism that shifts subpopulations of bacilli to dormancy [101]. Several *in vitro* models have been developed thus far to mimic these conditions of hypoxia and nutrient starvation [6,102,103]. Studies have shown that the NRP state brings about phenotypic resistance to anti-tuberculous agents, contributing to the challenge of effective disease control [104]. Ofloxacin and the sulbactam-ampicillin combination, for example, have shown reduced activity on stationary-phase cultures of *M. tuberculosis* [105]. While rifampicin, streptomycin, moxifloxacin and isoniazid are highly bactericidal for actively-replicating *M. tuberculosis*, they have little or no effect on the viability of nutrient-starved cultures [87,102]. The MBC_90_ of rifampicin increased 50 and 2,500 times under conditions of oxygen- and nutrient-starvation respectively, and isoniazid’s cidal activity was completely lost on *M. tuberculosis* cultured under both conditions for concentrations up to 100 µM [103]. In another study where 17 agents were tested against nutrient-starved *M. tuberculosis* at concentrations up to 160 µM, only four were able to achieve 99% killing (MBC_99_], and only when considerably higher test concentrations were used than against growing bacteria [106].

It has been suggested that, because anti-tuberculous agents typically target functions essential for growth and replication, they are less effective at eradicating NRP tuberculosis [101]. However, it is also believed that NRP bacilli develop alterations in their cell wall that affect permeability to antibiotics. Ziehl-Neelsen staining of *M. tuberculosis* in lung sections gradually fades with persistence of an infection [107]. The progression to the Ziehl-Neelsen-negative state is the result of cell wall composition alterations upon the onset of dormancy. Though loss of acid fastness has been associated with dormancy and phenotypic drug tolerance, the mechanisms by which such alterations mediate loss of acid fastness have not been elucidated [107].

NRP conditions lead to induction of the ‘dormancy regulon’, a collection of at least 48 genes that are controlled by the dormancy survival regulator DosR [108,109,110]. Under conditions of oxygen starvation, *M. tuberculosis* displays activation of several transport mechanisms including the up-regulated expression of predicted transporters for metal cations (*ctpA* and *ctpV*), sulphate (*Rv1739c*, *cysW*), molybdate (*modA*) and peptides (*dppA*) [111]. In a separate study, nutrient starvation caused phosphate uptake proteins (PstA1, PstB, PhoS1 and PstA2) to be down-regulated, and sulphate transport system proteins (CysA, CysW, CysT and SubI) to be up-regulated [102]. The regulation of expression of efflux pumps with antibiotic substrates under NRP conditions is still unclear. However, it is understood that the inactivation of Tap (Rv1258c), a known tetracycline efflux pump [74], in *M. bovis* BCG during stationary phase triggers a stress response that leads to a downshift in cell wall biosynthesis because of the accumulation of an unknown toxic substrate. This emphasizes that Tap is essential for the maintenance of balanced physiological function in the late stationary phase and indicates the potential role for the efflux pump during latency [112].

In bacterial species where it has been extensively studied, the regulation of porin expression has proven to be a fine-tuned and complex phenomenon modulated by multiple factors [113]. It is tempting to hypothesize that reduced porin density may contribute to the development of phenotypic drug tolerance in *M. tuberculosis*. Ongoing studies by our group aim to understand how starvation conditions affect the intracellular concentrations of standard anti-tuberculosis drugs in quiescent *M. tuberculosis* bacilli. 

### 6.2. Cell Wall Thickening

Recent ground breaking studies using cryo-electron tomography (CET) have revealed that the mycobacterial outer membrane is a symmetrical bilayer and might be less thick than generally believed in actively growing *M. smegmatis* and *M. bovis* BCG [114,115]. Cell wall thickening of the bacilli upon the onset of dormancy has been suggested based on transmission electron microscopy (TEM) studies and would have important implications on the persistence of *M. tuberculosis*. Caution should be exercised when interpreting TEM images, since electron microscopy analyses of ultrathin sections are performed with specimens from which water had been removed, a prerequisite for electron microscopy observation at room temperature. Consequently, water-soluble molecules may aggregate and lipid molecules may be prone to extraction or rearrangement by organic solvents during dehydration. This said, comparative analysis by TEM of *M. bovis* BCG and *M. tuberculosis* cultured under aerobic, micro-aerobic and anaerobic conditions revealed significant homogenous thickening of the cell wall in non-replicating quiescent bacilli [116]. More recently, thickening of the cell wall was observed in anaerobically grown quiescent *M. tuberculosis*, this time using atomic force microscopy [117]. Though these observations await confirmation *in vitro* and *in vivo*, as well as in-depth biochemical and structural analysis, a reinforced cell wall may constitute an additional permeability barrier, at least for some drug classes. Because porin dimensions and channel lengths presumably remain static despite these external changes, porin channels may not be able to span the depth of the thickened cell wall, thereby causing reduced access to the channel entrance by small molecules. 

### 6.3. Intracellular M. tuberculosis

As is the case for many bacterial pathogens, *M. tuberculosis* is phagocytosed by macrophages via the process of endocytosis. Some strategies for surviving the hostile intracellular environment of macrophages include the inhibition of phagosome-lysosome fusion and the inhibition of phagosome acidification [118,119]. Efflux pump P55 plays a significant role in *M. bovis* replication and persistence in the macrophages. It is functionally connected to P27 of the same operon (IprG-Rv1410c). The *p27-p55* knock-out mutation in *M. bovis* severely compromises virulence and intracellular replication [85]. In a recent elegant study, induction of drug tolerance in intracellular mycobacteria was attributed to macrophage-induced bacterial efflux mechanisms [92]. By using *M. tuberculosis*-infected cultured macrophages and *M. marinum*-infected zebrafish, the authors have shown that drug-tolerant bacteria arose within individual macrophages soon after infection and prior to granuloma formation. In contrast with the prevalent dogma, this drug tolerance was associated with a replicating intracellular bacterial population rather than macrophage-induced stasis. The study further showed that bacterial efflux pumps such as rifampicin-specific Rv1258c were induced upon macrophage infection, mediating drug tolerance. Interestingly, this drug tolerance was found to be retained for a period of time after bacteria resumed extracellular growth. 

## 7. Accumulation of Selected Drugs in *M. tuberculosis*

In the hunt for novel anti-tuberculosis drugs, the physico-chemical properties driving cell wall permeation by chemotherapeutic agents remain a critical but largely unsolved question. Table 4 lists biological and molecular properties of selected anti-TB agents, compiled in an attempt to detect correlation trends between intracellular accumulation and physico-chemical properties. The level of drug accumulation within *M. tuberculosis* cells varies significantly between drug classes but drawing comparisons between them is difficult due to differences in experimental methods used to measure accumulation. Intracellular concentrations of the fluoroquinolones listed were determined by measuring their fluorescence in cell lysates [120]. Pyrazinamide, isoniazid and rifampicin intracellular concentrations were calculated from scintillation counts of radio-labeled compounds in whole-cell preparations [121,122,123], which includes cell wall-associated drug content. This results in a possible overestimation of intracellular drug concentration. In the study of efficacy of drugs targeting cytosol-localized proteins, only the drug content of the cytosol compartment is of concern.

Molecular weight is often speculated to be an important determinant of the rate of diffusion and cell wall permeation; the smaller the compound the higher the rate of passive diffusion [124], while ClogP, a measure of the lipophilicity of a compound, reflects partitioning into the hydrophobic phase of the cell wall [125]. Attempts to plot these parameters as a function of intra-bacillary accumulation failed to reveal any correlations (not shown), consistent with the growing realization that many anti-TB agents lie outside the drug-like chemical space in which the Lipinski rule-of-five prevails [124,126]. The observation that the hydrophilic fluoroquinolones efficiently accumulate within *M. tuberculosis* supports the hypothesis that diffusion through porins serves as a route of transport across the mycobacterial cell wall for this class of compounds. Altogether, weak correlations are very likely due to the fact that many of these anti-tuberculous agents are transported via a combination of pathways. This analysis is also limited by the small number of drugs included and the fact that we attempted to compare physico-chemical properties across different compound classes. The intracellular concentrations of pro-drugs that require enzymatic activation are not always reflective of their potency; the intracellular concentration of active metabolites may be more relevant in the cases of isoniazid and pyrazinamide. 

Pyrazinamide appears to accumulate 6-fold within the bacilli, partly owing to its bioconversion to pyrazinoic acid and subsequent trapping of the charged molecule. Its transport has been determined as being ATP-dependent and reliant on the nicotinamide transport pathway [121]. However, the other drugs listed that accumulate intracellularly above the extracellular concentration have been speculated as being passively taken up by mycobacteria. In the case of isoniazid which accumulates 4–5 times within *M. tuberculosis*, the calculated accumulation factor accounts for both isoniazid and metabolites since the read-out measures all isoniazid-derived radioactive material. Constant conversion by KatG ensures a consistent pro-drug concentration gradient between the intracellular and extracellular compartments [122]. Fluoroquinolones appear to concentrate within the intracellular compartment despite being un-metabolized. The ability to concentrate a drug within the intracellular environment of *M. tuberculosis* should reflect the presence of active drug importers which have yet to be identified. 

In conclusion, there is no simple formula linking physico-chemical parameters to intracellular accumulation of small molecules in *M. tuberculosis*. As mentioned above, the contribution of a variety of passive and active mechanisms of uptake and efflux precludes the use of a simple equation to solve this question. Further studies focusing on relatively large numbers of molecules within the same chemical scaffold should help identify the major determinants of uptake for a given class of small molecules or therapeutic agents.

**Table 4:** Physico-chemical properties and intracellular accumulation factors of several antibiotics in *M. tuberculosis* as previously reported in published literature. Intracellular accumulation factors were defined as the ratio between intracellular and extracellular drug concentrations. Mechanisms of drug uptake were quoted or inferred from referenced publications.

Antibiotic	Molecular Weight	CLogP *	PSA (Å^2^) *	Target	IC_50_ (mg/L)	MIC_90_ (mg/L)	Accumulation Factor ^a^	Hypothesized Transport Mechanism	Reference
**Pyrazinamide**	123.12	−0.676	68.87	Fatty acid sysnthase I^b^	N.A.	16–50 (pH5.5)	5.4–6.2	ATP-dependent	[121,127]
**Isoniazid**	137.14	−0.668	68.01	Enoyl-acyl carrier protein reductase	N.A.	0.02–0.2	4–5	Passive Diffusion	[121,127]
**Ciprofloxacin**	331.35	−0.725	77.04	DNA Gyrase	3.2(*M. smeg*)	1.0	3.3–4.1	Passive Diffusion	[120,128]
**Levofloxacin**	361.38	−0.508	77.48	DNA Gyrase	3.0(*M. smeg*)	0.5	1.1–1.3	Passive Diffusion	[120,128]
**Ofloxacin**	361.38	−0.508	77.48	DNA Gyrase	7.9(*M. smeg*)	0.5	2.2–2.7	Passive Diffusion	[120,128]
**Norfloxacin**	319.34	−0.780	77.04	DNA Gyrase	Information unavailable	2	1.8–2.2	Passive Diffusion	[120]
**Moxifloxacin**	401.44	−0.082	86.27	DNA Gyrase	Information unavailable	0.5	1–1.3	Passive Diffusion	[120]
**Ethambutol**	204.32	0.119	64.52	Arabinosyl-transferase	Information unavailable	1– 5	<1	Passive Diffusion	[127,129]
**Rifampicin**	822.96	3.710	220.15	RNA polymerase	0.07(*M. avium*)	0.05–1	22.3–27.1	Passive Diffusion	[120]

^a ^Assuming cellular volume of 2.4–3.0µL per mg dry weight [121,122]; ^b^ See also work by Zhang *et al.* where an alternative mode of action for pyrazinamide is proposed [130]; * CLogP and PSA were calculated by the cheminformatics program *InSilico* Profile [131].

## 8. Future Perspectives

Thus far, tools have not been developed to accurately predict the extent to which a drug utilizes either the un-facilitated or porin-facilitated diffusion pathway to achieve cell wall penetration in mycobacteria. Several studies have looked at the complex inhibition of *E. coli* porins OmpF and OmpC by polyamines. Cadaverine, putrescine, spermidine and spermine have all been shown to inhibit both chemotaxis and the flux of β-lactam antibiotics in *E. coli* [132]. These polyamines have been shown to increase the number and duration of channel closures, while promoting the blocked or inactivated state [133,134]. The impact of polyamine inhibition on porins has not yet been demonstrated in mycobacteria but similar studies could open up the field of porin-mediated drug uptake in these species.

A recent elegant study by Allison *et al.* has shed light on the ability of specific metabolites to potentiate the eradication of both *E. coli* and *S. aureus* by aminoglycosides [135]. Aminoglycoside uptake in exponentially growing bacteria requires a PMF. Metabolites entering upper glycolysis such as glucose, mannitol and fructose enhance aminoglycoside uptake and the rapid killing of persisters by inducing PMF. It remains to be shown whether PMF-stimulating metabolites are similarly able to serve as adjuvants to aminoglycoside therapy for *M. tuberculosis* infections.

A handful of studies have established the link between antimicrobial drug uptake and active influx mechanisms in non-mycobacterial species. Streptomycin accumulation, for example, in *E. coli* and *P. aeruginosa* is energy-dependent and saturable [136]. Similar results were attained with gentamicin accumulation in the same two bacterial species; it was shown to concentrate between 4 to 250 times over the extracellular concentration [137]. It is conceivable that similar active drug influx mechanisms exist in *M. tuberculosis* and other mycobacterial species.

With regards to efflux pumps, the question remains: What chance is there of devising pump inhibitors as effective clinical solutions? Thioridazine appears to be a promising therapeutic option in the fight against MDR- and XDR-TB infections [138,139]. When used in combination with second-line anti-tuberculous agents it boosts the management of such infections [140]. Other than having been approved for use on the basis of compassionate therapy for XDR-TB patients in Mumbai, India, thioridazine remains untapped as an effective anti-tuberculous agent. Fellow anti-psychotic agent chlorpromazine has also displayed efflux pump inhibition activity in *M. avium* and *M. smegmatis* [138]. It is not known which specific class of bacterial efflux pumps is inhibited by these agents.

This review attempts to provide a holistic view of drug transport mechanisms in mycobacteria, and their potential contribution to intrinsic and acquired drug resistance, phenotypic drug tolerance, and genetic resistance. Improving our understanding in this field will help to decipher the virulence and resistance mechanisms of *M. tuberculosis*, and other pathogenic mycobacterial species.
